# Optimizing Chain Topology of Bottle Brush Copolymer for Promoting the Disorder-to-Order Transition

**DOI:** 10.3390/ijms23105374

**Published:** 2022-05-11

**Authors:** Jihoon Park, Hyun-Woo Shin, Joona Bang, June Huh

**Affiliations:** 1 Department of Chemical and Biological Engineering, Korea University, Seoul 02841, Korea; ayanami9306@korea.ac.kr; 2 College of Medicine, Seoul National University, Seoul 03080, Korea; charlie@snu.ac.kr; 3 Department of Life Sciences, Korea University, Seoul 02841, Korea

**Keywords:** block copolymer, bottle brush copolymer, order-disorder transition

## Abstract

The order-disorder transitions (ODT) of core-shell bottle brush copolymer and its structural isomers were investigated by dissipative particle dynamics simulations and theoretically by random phase approximation. Introducing a chain topology parameter λ which parametrizes linking points between *M* diblock chains each with *N* monomers, the degree of incompatibility at ODT (${\left(\chi N\right)}_{\mathrm{ODT}}$; χ being the Flory–Huggins interaction parameter between constituent monomers) was predicted as a function of chain topology parameter (λ) and the number of linked diblock chains per bottle brush copolymer (*M*). It was found that there exists an optimal chain topology about λ at which ${\left(\chi N\right)}_{\mathrm{ODT}}$ gets a minimum while the domain spacing remains nearly unchanged. The prediction provides a theoretical guideline for designing an optimal copolymer architecture capable of forming sub-10 nm periodic structures even with non-high χ components.

## 1. Introduction

Bottle brush copolymers (BBCs), where either copolymer side chain as a macromer or two or more kinds of side chains as comacromers are grafted densely to a linear polymer backbone, have attracted much interest owing to their intriguing phase behaviors which are similar but distinctively different from those of their linear counterpart [1,2,3,4,5,6,7,8,9,10,11]. In analogy with block copolymers with a linear chain topology, some BBCs in a molten state can form spatially periodic mesophases of which periodicities and phase transitions, such as order-disorder transitions, are strongly dependent upon the comonomer/comacromer sequence in the side/main chain of BBCs. For instance, it has been demonstrated that the periodicity of BBCs having two dissimilar types of homopolymer side chains (or macromer) with a blocky macromer sequence scales as $N^{0.9}$ where *N* is the degree of polymerization (DP) of the main chain, whereas that with a random macromer sequence is independent of *N* [6,7]. This rich behavior of BBC, owing to abundant options for constructing chain configurations, offers promising alternative means with a broader tunability of pattern dimension for developing patterning-related applications such as photonic crystals [4,12,13,14] and nanolithography [15,16] where the tuning of dimensions of the periodic structure is of critical importance.

Along with the periodicity, the order-disorder transition temperature (ODT), an indirect measure for the degree of segregation between unlike monomer species that constitutes a copolymer molecule, is one of the essential features that should be considered when designing copolymer nanostructures. As for linear block copolymers consisting only of two kinds of monomer species (viz. A and B), it is well-understood that the phase behavior is dictated by the degree of incompatibility, χN, where χ is the Flory–Huggins effective interaction parameter between the A and B monomer and *N* is, for this case, the DP of the unit building block (corresponding to $N=N_{A}+N_{B}$ where $N_{A}$ and $N_{B}$ are the DP of an A block and a B block, respectively) [17]. The phase separation occurs when the χN (=h) exceeds the value at ODT ($h_{\mathrm{ODT}}$), and the pitch of the periodic structure (*L*) scales as $L∼h^{1/6}N^{1/2}$ when $h\gg h_{\mathrm{ODT}}$ [18]. The value of $h_{\mathrm{ODT}}$ depends strongly on the chain architecture of BCPs, e.g., $h_{\mathrm{ODT}}$ = 10.495 for symmetric diblock [17,18], $h_{\mathrm{ODT}}$ = 17.996 for symmetric ABA triblock [19,20] in the mean-field limit (i.e., when *N* goes to infinity). This behavior has been a basic reference in the BCP lithography community who has been seeking novel BCP systems with a sub-10nm feature size. Previously, the sub-10 nm feature sizes of BCP have been achieved mainly by two approaches. One is to find BCPs with novel chemical pairs of A and B block where χ is high enough to ensure $h>h_{\mathrm{ODT}}$ even with small value of *N* (such that $L∼h^{1/6}N^{1/2}$ is still small) [21,22,23,24,25,26,27,28,29,30,31,32,33,34]. The second approach, which has been less reported, is to find BCPs with existing AB pairs but with nonconventional chain architectures that may lead to lower $h_{\mathrm{ODT}}$ (such that certain small values of $L∼h^{1/6}N^{1/2}$ still satisfies $h>h_{\mathrm{ODT}}$) [9,11,35,36]. As an example, a BBC with diblock side chains, often referred to as a core-shell bottle brush copolymer (CS-BBC), is known to exhibit the latter behavior. Previously, a theoretical work has shown that $h_{\mathrm{ODT}}$ is inversely related to the backbone DP of CS-BBC while the periodicity is asymptotically unchanged, which was also supported by an experimental work performed for the series of well-prepared CS-BBCs [10,11]. These works implicate that the phase separation can be promoted when the unit building blocks (diblocks) are linked to each other in such a way that the polymer architecture resembles the organization of building blocks in the phase-separated structure to cause the reduction of translational entropic loss associated with the formation of the ordered structure.

**Figure 1:**
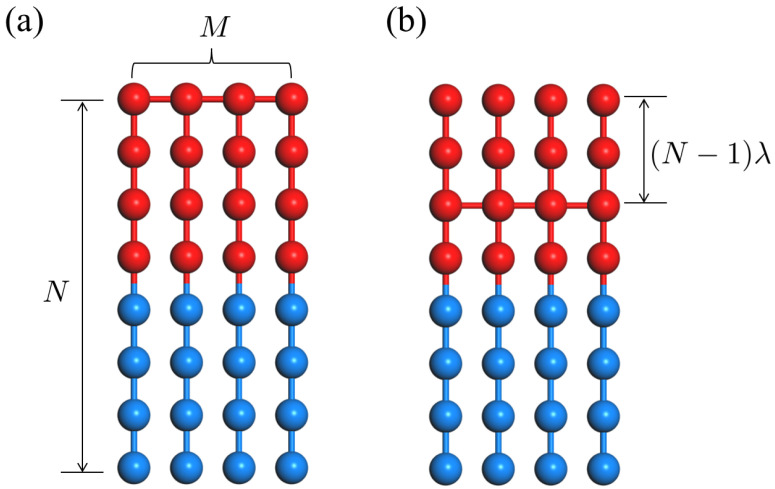
Illustration of polymer architectures consisting of *M* symmetric diblocks: (**a**) CS-BBC (λ=0); and (**b**) its structural isomers (λ>0).

This raises a further intriguing question of an optimization problem for chain topology: how unit building blocks should be linked to each other for the maximal promotion of phase separation while the periodicity is unchanged. Herein, we investigate the ODT behavior of some structural isomers of CS-BBC, where the structural variants are considered by varying the linking point between diblock chains along their chain paths (See Figure 1) using a dissipative particle dynamics (DPD) simulation and random phase approximation (RPA) theory.

## 2. Results and Discussion

We consider a general description for polymer architecture comprised of *M* symmetric diblocks connected somehow to each other where each symmetric diblock chain consists of *N* monomers. A CS-BBC chain and its structural isomers can be constructed by linking diblocks at the points parametrized by a fractional index λ which runs from 0 (the end of A-block) to 1 (the end of B-block), as depicted in Figure 1 (here only the case of λ<1/2 is considered without loss of generality). It is noted that M−N−λ space covers different chain topologies including simple diblock (M=1), triblock (M=2,λ=0), CS-BBC (M>1,λ=0), star copolymer with *M* diblock arms (M>1,N→∞,λ=0), miktoarm star copolymer with *M* arms (M>1,N→∞,λ=1/2), and many other structural isomers (M>1,λ>0).

For simulating molten states of these polymers, each having a given set of architectural parameters of {M,N,λ}, a dissipative particle dynamics (DPD) [37,38] is employed with the velocity-Verlet algorithm to time-integrate the equation of motions for A and B beads (monomers) that constitutes polymers with a given architecture. In DPD, the Flory–Huggins effective interaction parameter between the A and B monomer, χ, can be taken into account by $\Delta a=a_{AB}-\left(a_{AA}+a_{BB}\right)/2$ where $a_{ij}$ is the maximum repulsion between particle *i* and *j*, having the relation of $c\chi =\Delta a/k_{B}T$ where the density-dependent parameter *c* is given as c=3.27 for the present choice of bead density [38]. Each system was then equilibrated by stepwise-increasing $\Delta a/k_{B}T$ from an athermal state to a desired χ. The more detailed method is documented in the Simulation Methods.

**Figure 2:**
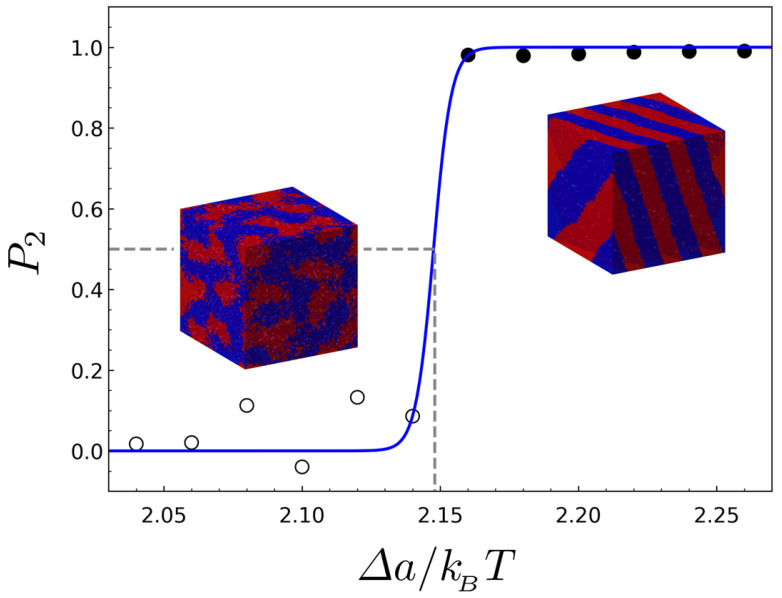
The order parameter versus the interaction parameter for the BBC having the architectural parameters of {M=8,N=14,λ=2/13}. The open circles and filled circles represent the points where the disordered and the ordered phases are stable, respectively, and the blue solid line is fit to a three-parameter sigmoidal function. The inset images show the two example structures simulated at the disordered and ordered region.

The ODT and domain spacing of the simulated systems are determined from scattering function given by

(1) $$\begin{array}{c} S\left(q\right)=\frac{1}{V}\langle \sum_{i<j}e^{iq\cdot (r_{i}-r_{j})}\Psi_{i}\Psi_{j}\rangle \end{array}$$

where q is the wave vector, *V* is the volume of the system, $r_{i}$ is the coordinates of the bead *i*, $\Psi_{i}$ is the occupation variable having the values of −1 or 1 if the bead *i* is an A bead or a B bead, respectively, and the bracket 〈〉 indicates a thermodynamic average. The choice of order parameter is of crucial importance in determining ODT [39,40]. In the present work, we choose the order parameter as a quantity related to the distribution of density fluctuations for A-monomer, which can be described by wave vectors q in the scattering function. When the ordered phase is formed, the order parameter must increase, implying that a certain wave vector becomes dominant. Considering the density fluctuation of A-monomer, we define the order parameter as the second-order coefficient of a series expansion of the orientation distribution ($P_{2}=\left(3\langle \cos^{2}\theta \rangle -1\right)/2$) where the orientation angle θ is interpreted as the angle between the wave vector q and a reference vector. The average value of $\cos^{2}\theta$, can be computed using the scattering function by

(2) $$\begin{array}{c} \langle \cos^{2}\theta \rangle =\frac{\sum_{q}{\left(\hat{q}\cdot {\hat{q}}_{1}\right)}^{2}S\left(q\right)}{\sum_{q}S\left(q\right)} \end{array}$$

where $\hat{q}$ and ${\hat{q}}_{1}$ are the unit vectors in the direction of a wave vector q and in the direction of the dominant wave vector $q_{1}$, respectively. Figure 2 exemplifies the determination of ODT by the order parameter that is plotted against Δa for {M=8,N=14,λ=2/13}.

The spinodal of the molten copolymer, which can be a reasonable approximation of ODT, was also theoretically determined by the random phase approximation (RPA) equation: $1/S\left(x\right)=\sum_{\alpha \beta }G_{\alpha \beta }\left(x\right)/\left|G_{\alpha \beta }\left(x\right)\right|-2\chi$ where $x=q^{2}R^{2}$ with *R* being the root mean square radius of gyration of a diblock and $G_{\alpha \beta }$ is the single chain density correlation function between monomer type α and β (α,β = A or B) in the ideal state. For the architecture of {M,N,λ} where λ≤1/2, $G_{\alpha \beta }$ is given as

(3) $$\begin{array}{cc} \; & G_{AA}\left(x\right)=Ng_{1}\left(1/2,x\right)+\frac{NS}{M}{\left[g_{2}\left(0,1/2-\lambda ,x\right)+g_{2}\left(0,\lambda ,x\right)\right]}^{2} \end{array}$$

(4) $$\begin{array}{cc} \; & G_{BB}\left(x\right)=Ng_{1}\left(1/2,x\right)+\frac{NS}{M}g_{2}^{2}\left(1/2-\lambda ,1/2,x\right) \end{array}$$

(5) $$\begin{array}{cc} \; & G_{AB}\left(x\right)=Ng_{2}^{2}\left(0,1/2,x\right)+\frac{NS}{M}g_{2}\left(1/2-\lambda ,1/2,x\right)\left[g_{2}\left(0,1/2-\lambda ,x\right)+g_{2}\left(0,\lambda ,x\right)\right] \end{array}$$

(6) $$\begin{array}{cc} \; & G_{BA}\left(x\right)=G_{AB}\left(x\right) \end{array}$$

where

(7) $$\begin{array}{cc} \; & g_{1}\left(f,x\right)=\frac{2}{x^{2}}\left(fx+e^{-fx}-1\right) \end{array}$$

(8) $$\begin{array}{cc} \; & g_{2}\left(f_{1},f_{2},x\right)=\frac{1}{x}\left(e^{-f_{1}x}\left(1-e^{-f_{2}x}\right)\right) \end{array}$$

(9) $$\begin{array}{cc} \; & S=\sum_{i}^{M}\sum_{j\neq i}^{M}e^{-\frac{x}{N}\left|i-j\right|} \end{array}$$

The formulas for λ>1/2 can be obtained by switching the monomer type A and B and changing λ to 1−λ in Equations (3)–(6). The first terms of $G_{\alpha \beta }$ in Equations (3)–(6) takes into account the monomer correlations within a side chain while the second term represents the correlation between different side chains. One can check that Equations (3)–(6) reduce to that for the diblock chain when M=1 (i.e., S=0, no inter-side chain correlations) and it reduces to that for the star copolymer when N=∞ (i.e., S=M(M−1)). The spinodal is then determined from the divergence of the scattering function at the dominant wave vector $x_{1}$.

**Figure 3:**
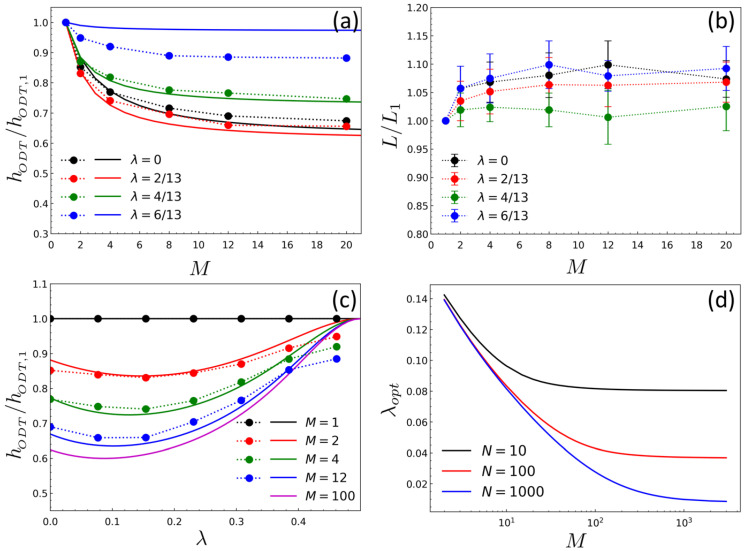
The behavior of ODT and domain spacing of BCPs with various chain topologies: (**a**) The normalized ODT ($h_{\mathrm{ODT}}/h_{\mathrm{ODT},1}$) as a function of the backbone length *M* for different topology parameter λ, where $h_{\mathrm{ODT},1}$ is $h_{\mathrm{ODT}}$ of diblock (M=1); (**b**) the normalized domain spacing ($L/L_{1}$) as a function of the backbone length *M* for different λ, where $L_{1}$ represents *L* at M=1; (**c**) The normalized ODT as a function of λ for different *M*; (**d**) The optimal value of topology parameter ($\lambda_{opt}$) as a function of *M* for different *N*. In (**a**–**d**), the symbols with dotted lines represent the DPD results and the solid lines represent the RPA results. All computations were obtained for the side chain length of N=14. The domain spacing shown in (**b**) were obtained at $\Delta a/k_{B}T=3.5$.

Figure 3a,b show the variation of ODT ($h_{\mathrm{ODT}}$) and the domain spacing (*L*), which are normalized by those of diblock case (M=1), as a function of the number of linked diblocks *M* for a different chain topology parameter λ. Despite relatively small chain molecules (M≤20,N=14) simulated in this study, the DPD results agree well with RPA results, both showing the same behavior that $h_{\mathrm{ODT}}$ decreases as *M* increases for all λ while $L_{o}$ is varied within 10% dilation when compared to that of the neat diblock chain. Of most interest in the ODT result is that there is an optimal value of λ ($\lambda_{opt}$) at which $h_{\mathrm{ODT}}$ attains a minimum. This behavior is analyzed more clearly in Figure 3c where $h_{\mathrm{ODT}}$ is plotted against λ for different *M*. As seen in Figure 3c, $\lambda_{opt}$ decreases as *M* increases, approaching an asymptotic value ($\lambda_{opt}≃0.086$ for N=14) when M→∞. It can be also shown from RPA that $\lambda_{opt}\to 0$ in the limiting case of M→∞ and N→∞ (Figure 3d). This topological effect on the ODT can be understood qualitatively by considering two opposing entropic changes upon the formation of the ordered phase: one is the entropy loss of side chains associated with confining AB-junctions of copolymers at the A/B interface, and the other is the entropic loss associated with restricting the backbone that has to be tethered by side chains to the interface. The former decreases as the backbone (or linking point) becomes closer to the A/B junction points (i.e., λ approaches 1/2), whereas the latter increases as λ→1/2, which leads to the optimal behavior of ODT with respect to λ.

**Figure 4:**
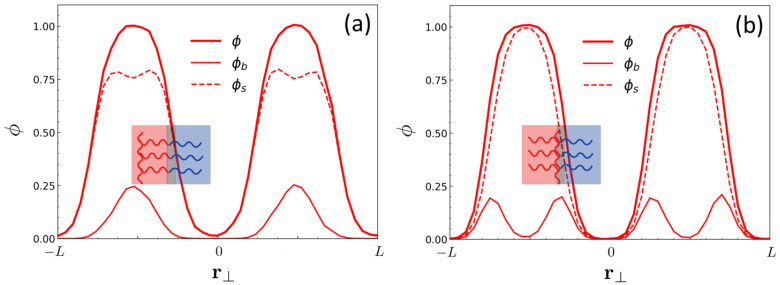
The local volume fraction of A-monomers (ϕ), that belonging to the backbone ($\phi_{b}$), and that belonging to the side chain ($\phi_{s}$) in the direction perpendicular to the lamellar interface ($r_{⊥}$) obtained by DPD simulations for (**a**) {M=12,N=14,λ=0} and for (**b**) {M=12,N=14,λ=6/13}. All profiles were obtained at $\Delta a/k_{B}T=3.5$. The pictures in the inset show the schematic representations of BBC organization in the lamellar phase with a half pitch for each case.

In Figure 4, the molecular packing of copolymers with {M=12 and N=14} in the lamellar phase for two contrasting cases, λ=0 and λ=6/13, are compared by plotting the local volume fraction of A-monomers in the direction perpendicular to the lamellar interface, $\phi (r_{⊥})$, and its decomposed components, the volume fraction of the A-monomer belonging to the backbone, $\phi_{b}\left(r_{⊥}\right)$, and that belonging to the side chain, $\phi_{s}\left(r_{⊥}\right)$. As expected, the volume fraction profiles show that in the case of chain topology with λ=0 where the linking points are located at the diblock ends (i.e., CSBB), the backbones are found around the center of the A-phase, whereas in the case of λ=6/13 close to the A-B junction point (λ=1/2) the backbones are populated near the A/B interfaces. Wider distribution of $\phi_{b}\left(r_{⊥}\right)$ over the entire A-domain in the case of λ=0 reflects less restriction on backbone conformation as compared to the case of λ≃1/2, which cause the promoted ODT.

## 3. Simulation Methods

All simulations were performed by a dissipative particle dynamics (DPD) [37,38] with HOOMD package [41]. In DPD where polymers in a coarse-grained description are modeled by bead-spring chains [42,43], each bead representing a Kuhnian segment is modeled to interact with each other via a simple force which is a pairwise additive. The force $f_{i}$ acting on bead *i* of mass $m_{i}$ at a position vector $r_{i}$ consists of

(10) $$\begin{array}{c} f_{i}=m_{i}{\ddot{r}}_{i}=\sum_{j\neq i}\left(F_{ij}^{\left(C\right)}+F_{ij}^{\left(D\right)}+F_{ij}^{\left(R\right)}+F_{ij}^{\left(S\right)}\right) \end{array}$$

where $F_{ij}^{\left(C\right)}$, $F_{ij}^{\left(D\right)}$, $F_{ij}^{\left(R\right)}$, and $F_{ij}^{\left(S\right)}$ are a conservative force, a drag force, a random force, and spring force between bead *i* and *j*, respectively. The conservative force $F_{ij}^{\left(C\right)}$ is a soft core repulsion, given by

(11) $$\begin{array}{c} F_{ij}^{\left(C\right)}=\left\{\begin{array}{cc} a_{ij}\left(1-\frac{r_{ij}}{R_{c}}\right){\hat{r}}_{ij} & \mathrm{for}\;r_{ij}<R_{c}\\ 0 & \mathrm{otherwise} \end{array}\right. \end{array}$$

where $a_{ij}$ is a maximum repulsion ($a_{ij}>0$) between between bead *i* and *j*, $r_{ij}$ is the distance between bead *i* and *j*, ${\hat{r}}_{ij}$ is a unit vector along the direction from bead *i* to bead *j*, i.e., ${\hat{r}}_{ij}=\left(r_{j}-r_{i}\right)/\left|r_{j}-r_{i}\right|$, and $R_{c}$ is the cutoff distance. The drag force $F_{ij}^{\left(D\right)}$ and the random force $F_{ij}^{\left(R\right)}$ have the forms of

(12) $$\begin{array}{cc} \; & F_{ij}^{\left(D\right)}=-\gamma {\left[w(r_{ij})\right]}^{2}\left({\hat{r}}_{ij}\cdot {\dot{r}}_{ij}\right){\hat{r}}_{ij} \end{array}$$

(13) $$\begin{array}{cc} \; & F_{ij}^{\left(R\right)}=\zeta_{ij}\left(t\right)w\left(r_{ij}\right)\sqrt{\frac{6k_{B}T\gamma }{\delta t}}{\hat{r}}_{ij} \end{array}$$

Here, γ is the friction coefficient, $w(r_{ij})$ is a weight function related to $r_{ij}$, $\zeta_{ij}$ is a random number uniformly distributed in the range of [−1, 1] generated independently for each pair of bead *i* and *j* at each time step, $k_{B}T$ is thermal energy, and δt is the time step size. Consistency between kinetic energy and thermal energy are ensured in Equations (12) and (13) via the amplitude of random noise ($\sqrt{6k_{B}T\gamma /\delta t}$) [37,44]. Furthermore, the weight function *w* is chosen to have the following form,

(14) $$\begin{array}{c} w\left(r\right)=\left\{\begin{array}{cc} 1-\frac{r}{R_{c}} & \mathrm{for}\;r<R_{c}\\ 0 & \mathrm{otherwise} \end{array}\right. \end{array}$$

Lastly, the bonding between bead *i* and *j*, responsible for chain connectivity, is taken into account by a spring force, $F_{ij}^{\left(S\right)}$,

(15) $$\begin{array}{c} F_{ij}^{\left(S\right)}=-K\left(r_{ij}-r_{o}\right){\hat{r}}_{ij} \end{array}$$

where *K* is the spring constant and $r_{o}$ is the equilibrium bond length. The equation of motions Equation (10) for beads in the system were time-integrated using the velocity-Verlet algorithm [45]:

(16) $$\begin{array}{cc} \; & r_{i}\left(t+\delta t\right)=r_{i}\left(t\right)+{\dot{r}}_{i}\left(t\right)\delta t+\frac{1}{2}{\ddot{r}}_{i}\left(t\right)\delta t^{2} \end{array}$$

(17) $$\begin{array}{cc} \; & {\dot{r}}_{i}\left(t+\delta t\right)={\dot{r}}_{i}\left(t\right)+\frac{1}{2}{\ddot{r}}_{i}\left(t\right)\delta t+\frac{1}{2}{\ddot{r}}_{i}\left(t+\delta t\right)\delta t \end{array}$$

The basic units for length, mass, energy, and time in the simulation are set to be $R_{c}=1$, m=1, $k_{B}T=1$, and $t=R_{c}\sqrt{m/k_{B}T}=1$, respectively, and the time step δt is set to be δt=0.01 which is specified from the unit thermal energy, $k_{B}T=1$, for the consistency between thermal and kinetic energy. All DPD parameters introduced Equations (11)–(15) are rescaled according to these basic units, which are listed in Table 1.

Using the bead-spring chain model by DPD, architecturally monodisperse BBCs, each of which has a given chain architecture characterized by a set of parameters {M,N,λ}, were generated in a $30R_{c}\times 30R_{c}\times 30R_{c}$ simulation box with a number density of beads $\rho =3R_{c}^{-3}$ chosen for the molten state. The periodic boundary conditions were applied in all axes of the simulation box. The unfavorable interaction between the bead of type A and type B was modeled using the maximum repulsion $a_{ij}$ introduced in Equation (11), whose value is given from the Flory interaction parameter between A- and B-bead, χ, using the relation, $c\chi =\Delta a/k_{B}T$, where $\Delta a=a_{AB}-\left(a_{AA}+a_{BB}\right)/2$ and the density-dependent parameter *c* is given as c=3.27 for the present choice of bead density [38]. The maximum repulsion between the same kind of beads is set to be $a_{AA}=a_{BB}=25k_{B}T/R_{c}$ and that between A and B beads, $a_{AB}$, is given according to a desired χ. The ordered state of each system was obtained by stepwise-increasing $\Delta a/k_{B}T$ from an athermal state ($\Delta a/k_{B}T=0.0$) to $\Delta a/k_{B}T=3.5$ with an increment of $\Delta (\Delta a/k_{B}T)=0.5$. Having obtained the ordered structures at $\Delta a/k_{B}T=3.5$, each ordered system was then slowly annealed to a desired $\Delta a/k_{B}T$ by stepwise-decreasing $\Delta a/k_{B}T$ with a very small decrement of $\Delta (\Delta a/k_{B}T)=0.02$ where the system was equilibrated at each of $\Delta a/k_{B}T$ for $1.5\times 10^{6}\delta t$ followed by the production step for $5\times 10^{5}\delta t$ to produce configuration samples for thermodynamic average.

## 4. Conclusions

In conclusion, we theoretically investigated the effect of chain topology on the ODT of a series of BBC where *M* diblocks each with *N* monomers are linked to each other at the linking point parametrized by a chain topology parameter λ. It is found from DPD simulation and RPA approach that the degree of incompatibility χN at ODT ($h_{\mathrm{ODT}}$) of the BBC decreases as *M* increases for all cases of λ while the domain spacing is varied within 10% dilation. For instance, a CSBB having a backbone with M=100 and side chains with N=10 has an ODT approximately at χN=6.2, a 60 % reduction as compared to that of diblock (M=1). This implies that the limiting minimum of the domain spacing of BBC, associated with the minimal molecular weight $N_{min}=h_{\mathrm{ODT}}/\chi$ for a given monomer pair (i.e., fixed χ), is smaller than that of diblock. We also found that there exists an optimal topology about λ ($\lambda_{opt}$) at which $h_{\mathrm{ODT}}$ gets a minimum, and obtained that $\lambda_{opt}<0.16$ for M>2 and $\lambda_{opt}\to 0$ as M→∞ and N→∞. While a chain topology with 0<λ<0.16 seems synthetically challenging, an approximate chain topology for such BBCs can be achieved in practice by a random sequence of diblock and homopolymer chains as comacromers (see, for instance, the BBC architecture in graphical abstract). The finding here provides a theoretical guideline for designing a copolymer architecture capable of forming sub-10nm periodic structures even with non-high χ components. The ODT analysis in the present work is limited to the lamellar phase and the extension to the architecture effect of topological parameter on the non-lamellar phase will be future works.

## Figures and Tables

**Table 1:** The list of the DPD parameters used in the present study.

Parameter	Value	Unit ^1^	Equations
$a_{ii}\;\left(a_{AA},a_{BB}\right)$	25.0	$k_{B}T/R_{c}$	(11)
γ	4.5	$\sqrt{mk_{B}T}/R_{c}$	(12), (13)
*K*	100.0	$k_{B}T/R_{c}^{2}$	(15)
$r_{o}$	1.5	$R_{c}$	(15)

^1^ The basic units for length, mass, and the energy are set to be $R_{c}$ = *1*, *m* = 1, and $k_{B}T$ = 1, respectively.

## Data Availability

The data presented in this study are available on request from the corresponding author.
